# Supplementary material for: Blastocyst complementation using Prdm14-deficient rats enables efficient germline transmission and generation of functional mouse spermatids in rats
Source: Nat Commun. 2021 Feb 26;12:1328. doi: 10.1038/s41467-021-21557-x (PMC7910474; doi:10.1038/s41467-021-21557-x)
Supplement: Supplementary file 1 — Supplementary information [file 41467_2021_21557_MOESM1_ESM.pdf]

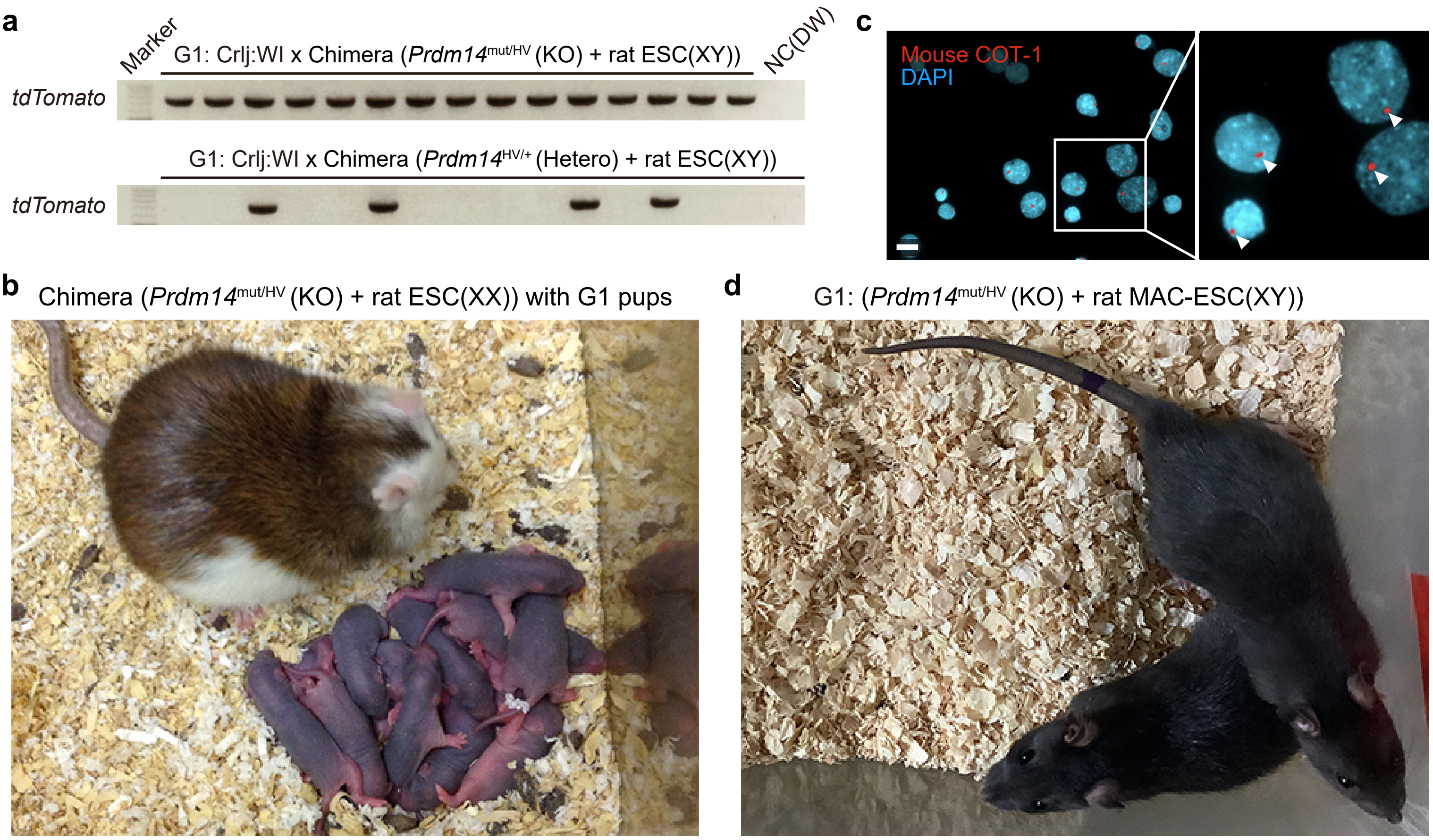

Supplementary Figure 1 Germline transmission of donor rat ESCs. **a** A representative genotyping result to detect the *Rosa26-tdTomato* transgene. **b** FISH image of brain cells from rat carrying MAC generated from rat ESCs. Scale bars: 10 μm. **c** A female *Prdm14*$^{mut/HV}$ chimeric rat generated by injection of *Rosa26*$^{tdTomato/tdTomato}$ rat ESCs (XX) with its offspring. All the neonates showed black eye/coat color indicating complete germline transmission of donor ESCs. **d** Adult rats obtained by ROSI of MAC containing rat ESC -derived spermatids into rat oocytes. Black eye/coat color originates from donor ESCs.

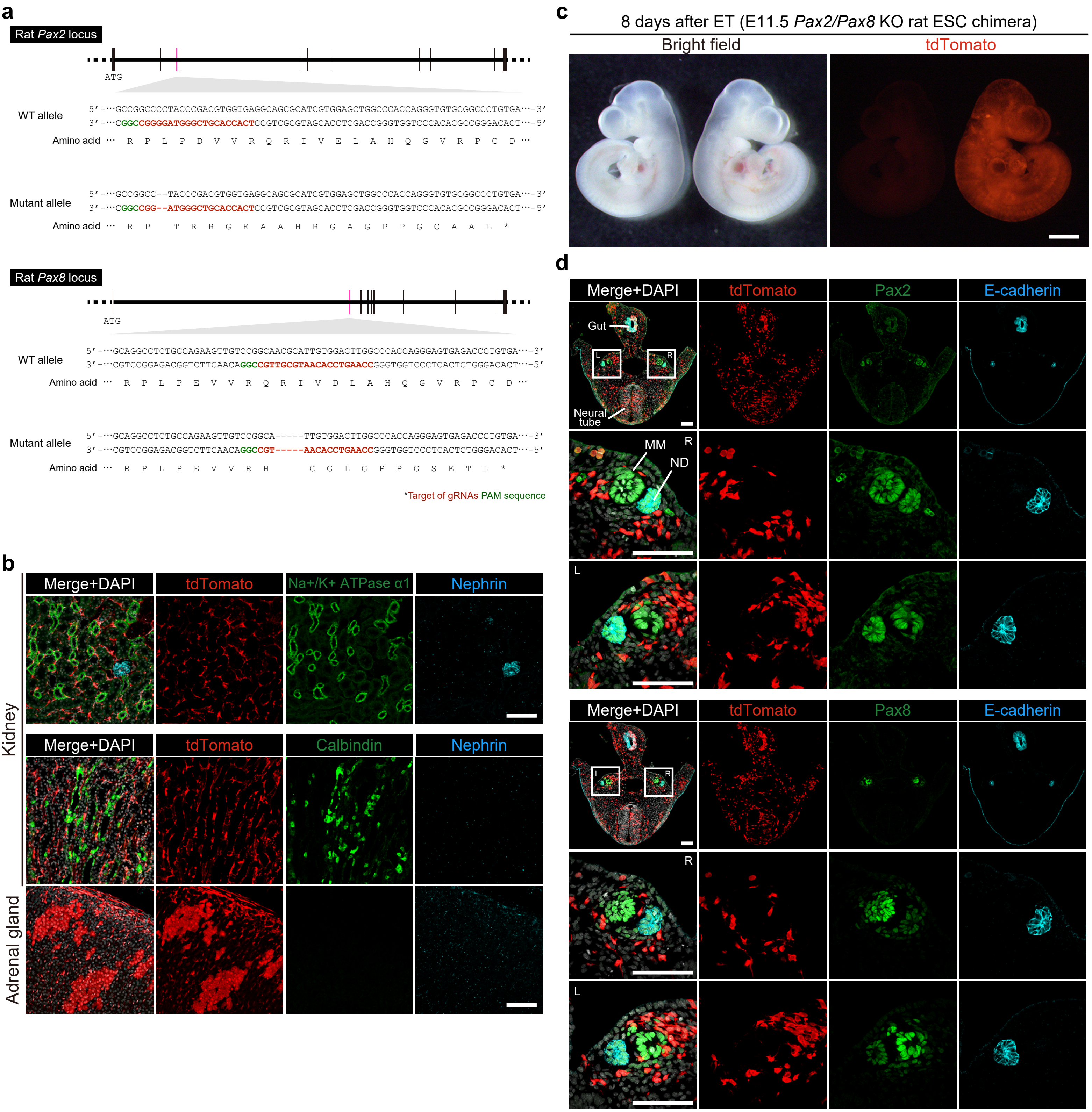

Supplementary Figure 2 ***Pax2/Pax8* KO rat ESCs and their contribution to chimeras.** **a** Sequences of *Pax2* and *Pax8* alleles in double mutant rat ESCs. **b** Immunohistochemical staining of kidney and adrenal grand in *Prdm14*$^{mut/HV}$ chimeric rats with *Pax2/Pax8* KO rat ESCs. As in **Fig. 2b**, all renal lineages (Na+/K+ ATPase α1 positive distal tube, Calbindin positive collecting duct, and Nephrin positive glomeruli) originated from host embryos. The adrenal grand, as a control for non-renal and gonadal tissue, was composed of both donor and host-derived cells. Scale bar: 100 μm. **c** Representative chimeric fetus (right) at 8 days after embryo transfer (ET: embryonic day (E) 11-11.5) generated by injection of *Pax2/Pax8* KO rat ESCs into rat blastocyst. Non-chimera (left) is shown as a control. Scale bar: 1 mm. **d** Immunohistochemical staining of chimeric fetus generated in **Supplementary Figure 2c**. Metanephric mesenchyme (MM) and E-cadherin positive nephric duct (ND) are shown. Scale bars: 100 μm.

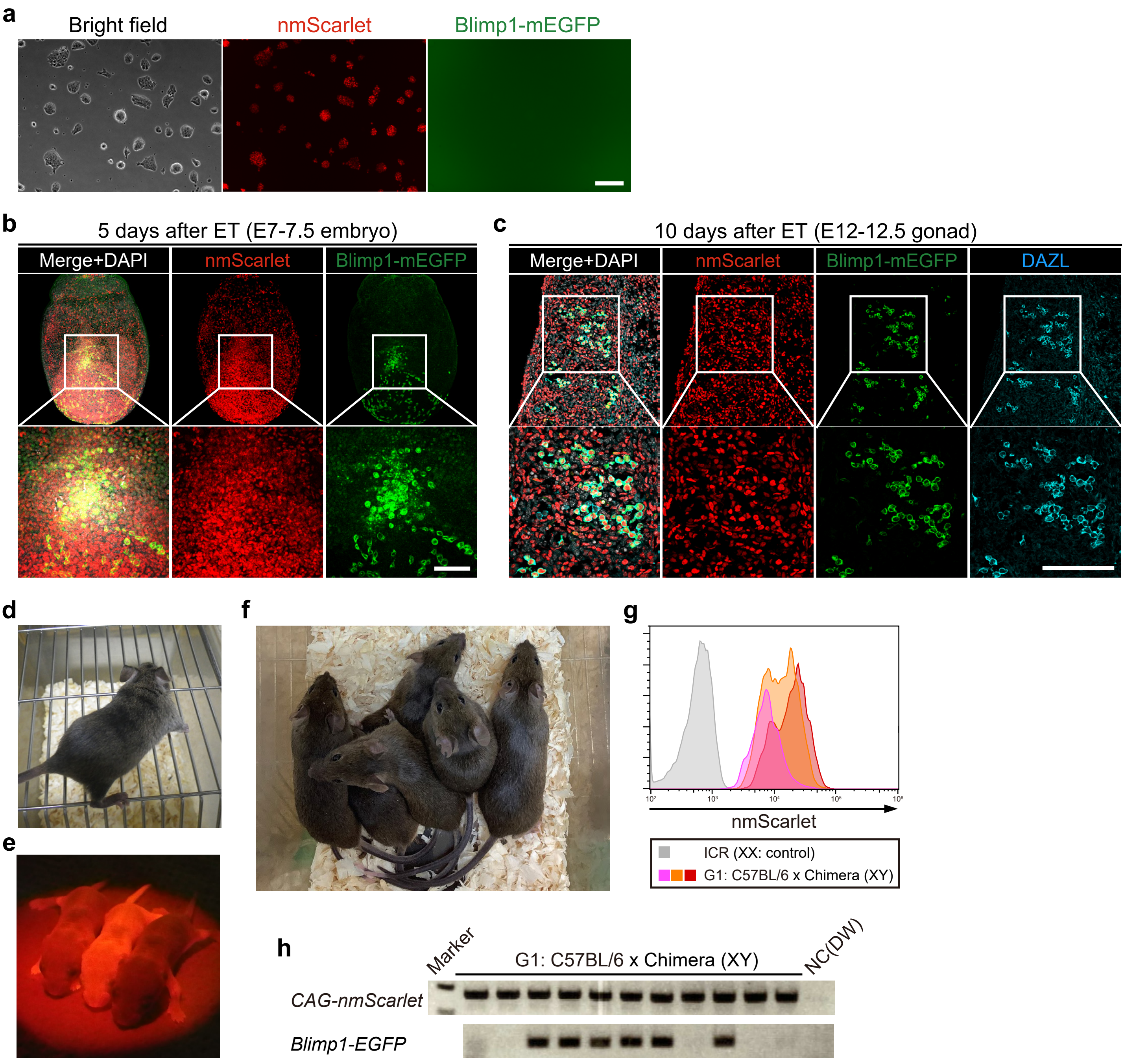

Supplementary Figure 3 Characterization of *Blimp1*$^{Tg(BAC-mEGFP)/+}$*/CAG-nmScarlet* mouse ESCs.
a A representative image of undifferentiated mouse ESCs ubiquitously expressing nmScarlet but not mEGFP. Scale bar: 200 μm. **b** Wholemount immunostaining of posterior part of a chimeric mouse embryo with mouse ESCs at 5 days after embryo transfer (ET: embryonic day (E) 7-7.5). Mouse ESCs efficiently contributed both somatic cells and Blimp1-mEGFP positive PGCs. Scale bar: 100 μm.
c Immunohistochemical staining of a male mouse chimeric gonad at 10 days after ET (E12-12.5). mEGFP/nmScarlet/DAZL triple positive mouse ESC-derived PGCs were observed. Scale bar: 100 μm.
d A representative image of adult chimeric mouse. The agouti coat color originated from injected mouse ESCs. **e** Neonates obtained by crossing chimeric mouse with wild type C57BL/6NCrSlc mice. While the expression level varied, all the neonates in the images expressed nmScarlet ubiquitously, suggesting successful germline transmission. **f** Adult mice showing dark agouti coat color originating from donor mouse ESCs. **g** Representative FACS patterns of peripheral blood in adult mice derived from mouse ESCs. Expression patterns of nmScarlet varied depending on the mice, which is likely due to random insertion of the transgene via PiggyBac system. **h** Genotyping showing detection of *CAG-nmScarlet* and *Blimp1-mEGFP* transgenes in adult mice derived from mouse ESCs.

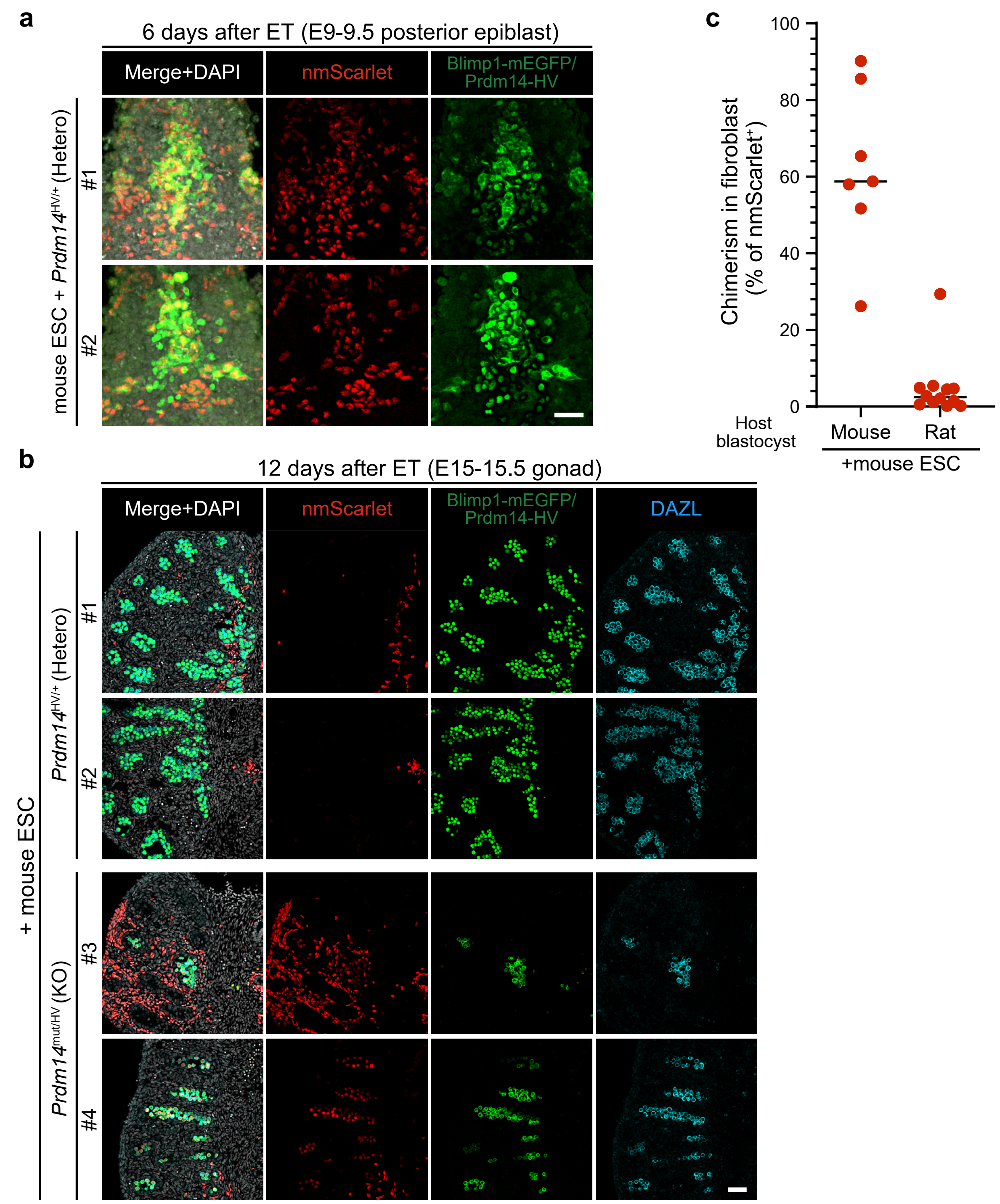

Supplementary Figure 4 Analysis of interspecific chimera generated by injection of mouse ESCs into rat blastocysts. a Wholemount immunostaining of posterior part of *Prdm14*$^{HV/+}$ (Heterozygous) chimeric embryo with mouse ESCs at 6 days after embryo transfer (ET: embryonic day (E) 9-9.5). Both chimeras showed presence of mEGFP/nmScarlet double positive mouse PGCs with Prdm14-H2BVenus rat PGCs. Scale bar: 50 μm. **b** Immunohistochemical staining of male gonads of *Prdm14*$^{mut/HV}$ and *Prdm14*$^{HV/+}$ chimeric fetus at 12 days after ET (E15-15.5). mEGFP/nmScarlet/DAZL triple positive mouse PGCs colonized in rat gonad. No mouse PGCs were observed in *Prdm14*$^{HV/+}$ chimeric fetus with rat PGCs. Scale bar: 50 μm. **c** Dot plots show chimerism of mouse ESCs in embryonic fibroblast after injection into mouse (left) or rat (right) blastocysts, respectively. The embryonic fibroblasts were established from the fetus (E12.5 in mouse [n=7], E15.5 in rat [n=12]).

ID	Gender of donor ESC	Host embryo		No. of pregnancy	No.(%) of pups		Blood chimerism (% of tdTomato+)
		Gender	*Prdm14* genotype		Analyzed	tdTomato+	
2373-Ep11*	XY	XY	KO	3	46	46 (100)	96.8
2380-Ep19				0	ND	ND	0.10
2381-Ep24				3	39	39 (100)	2.33
			Total	6	85	85 (100)	
2388-Ep38*	XX	XX	KO	1	14	14 (100)	76.8
2388-Ep46				0	ND	ND	3.54
2409-Ep71				1	12	12 (100)	91.1
2409-E72				1	5	5 (100)	58.2
			Total	3	31	31 (100)	
2373-Ep8*	XY	XY	Hetero	2	22	6 (27)	93.4
2380-Ep20				3	43	0 (0)	0.00
2381-Ep23				3	41	8 (20)	91.0
2381-Ep28				2	26	1 (3.8)	96.0
2381-Ep29				3	42	1 (2.4)	93.0
			Total	13	174	16 (9.2)	
2371-Ep6	XX	XX	Hetero	1	15	0 (0)	83.3
2388-Ep43				1	13	0 (0)	74.7
2388-Ep44				1	13	3 (23)	88.4
2388-Ep45				1	17	6 (35)	86.3
2388-Ep39				1	14	0 (0)	0.02
2409-Ep73*				1	15	1 (6.7)	73.5
			Total	6	87	10 (11)	

*Shown in **Fig.1** as representative chimeras

Supplementary Table 1 List of chimeric rats with *Rosa26*$^{tdTomato/tdTomato}$ rat ESCs used for progeny test.

Antigen	Company	Cat No.	Dilution
GFP	Abcam	ab13970	1:500
DsRed	Takara	632496	1:500
mCherry	EnCor	CPCA-mCherry	1:500
DAZL	NOVUSBIO	NBP2-61937	1:250
DDX4	Abcam	ab27591	1:400
SOX9	Abcam	ab186966	1:500
FOXL2	Abcam	ab5096	1:200
Aquaporin1	Millipore	AB2219	1:50
Na+/K+ ATP ase α-1	Millipore	05-369	1:50
Calbindin	Abcam	ab82812	1:250
Nephrin	R&D SYSTEMS	AF3159	1:250
Pax2	BioLegend	901001	1:100
Pax8	Abcam	ab189249	1:500
E-cadherin	BD	BD610181	1:250

Supplementary Table 2 List of antibodies used for immunohistochemical staining
