# Supplementary material for: Blastocyst complementation using Prdm14-deficient rats enables efficient germline transmission and generation of functional mouse spermatids in rats
Source: Nat Commun. 2021 Feb 26;12:1328. doi: 10.1038/s41467-021-21557-x (PMC7910474; doi:10.1038/s41467-021-21557-x)
Supplement: Supplementary file 3 — Reporting Summary [file 41467_2021_21557_MOESM3_ESM.pdf]

nature research

Corresponding author(s): Toshihiro Kobayashi, Masumi Hirabayashi

Last updated by author(s): Jan 20, 2021

Reporting Summary

Nature Research wishes to improve the reproducibility of the work that we publish. This form provides structure for consistency and transparency in reporting. For further information on Nature Research policies, see our Editorial Policies and the Editorial Policy Checklist.

Statistics

For all statistical analyses, confirm that the following items are present in the figure legend, table legend, main text, or Methods section.

n/a	Confirmed	
☐	☒	The exact sample size (n) for each experimental group/condition, given as a discrete number and unit of measurement
☐	☒	A statement on whether measurements were taken from distinct samples or whether the same sample was measured repeatedly
☒	☐	The statistical test(s) used AND whether they are one- or two-sided<br>*Only common tests should be described solely by name; describe more complex techniques in the Methods section.*
☒	☐	A description of all covariates tested
☒	☐	A description of any assumptions or corrections, such as tests of normality and adjustment for multiple comparisons
☒	☐	A full description of the statistical parameters including central tendency (e.g. means) or other basic estimates (e.g. regression coefficient) AND variation (e.g. standard deviation) or associated estimates of uncertainty (e.g. confidence intervals)
☒	☐	For null hypothesis testing, the test statistic (e.g. F, t, r) with confidence intervals, effect sizes, degrees of freedom and P value noted<br>*Give P values as exact values whenever suitable.*
☒	☐	For Bayesian analysis, information on the choice of priors and Markov chain Monte Carlo settings
☒	☐	For hierarchical and complex designs, identification of the appropriate level for tests and full reporting of outcomes
☒	☐	Estimates of effect sizes (e.g. Cohen's d, Pearson's r), indicating how they were calculated

Our web collection on statistics for biologists contains articles on many of the points above.

Software and code

Policy information about availability of computer code

Data collection	SH800 software for FACS analysis.
Data analysis	FlowJo v10 for FACS data analysis. FV31S-DT for LSM images. Adobe photoshop for editing and formatting the images for the figures.

For manuscripts utilizing custom algorithms or software that are central to the research but not yet described in published literature, software must be made available to editors and reviewers. We strongly encourage code deposition in a community repository (e.g. GitHub). See the Nature Research guidelines for submitting code & software for further information.

Data

Policy information about availability of data

All manuscripts must include a data availability statement. This statement should provide the following information, where applicable:

- Accession codes, unique identifiers, or web links for publicly available datasets
- A list of figures that have associated raw data
- A description of any restrictions on data availability

All the data in this study are available from the corresponding author upon reasonable request.

Field-specific reporting

Life sciences study design

All studies must disclose on these points even when the disclosure is negative.

Sample size	Sample size was depended on genotype, animals and chimeras generated by embryo manipulations. The details of the embryo manipulation are listed in Table 1 and 2.
Data exclusions	No data were excluded from the analyses.
Replication	All attempts at replication were successful in this study.
Randomization	n/a
Blinding	n/a

Reporting for specific materials, systems and methods

We require information from authors about some types of materials, experimental systems and methods used in many studies. Here, indicate whether each material, system or method listed is relevant to your study. If you are not sure if a list item applies to your research, read the appropriate section before selecting a response.

Materials & experimental systems

n/a	Involved in the study	
☐	☒	Antibodies
☐	☒	Eukaryotic cell lines
☒	☐	Palaeontology and archaeology
☐	☒	Animals and other organisms
☒	☐	Human research participants
☒	☐	Clinical data
☒	☐	Dual use research of concern

Methods

n/a	Involved in the study	
☒	☐	ChIP-seq
☐	☒	Flow cytometry
☒	☐	MRI-based neuroimaging

Antibodies

Antibodies used	All the antibodies used in this study are listed in Supplementary Table 2.
Validation	All primary antibodies were validated by the supplier and us as listed in Supplementary Table 2.

Eukaryotic cell lines

Policy information about cell lines

Cell line source(s)	Described in "Culture of pluripotent stem cell lines" in the Methods section.
Authentication	Described in the Methods section.
Mycoplasma contamination	All the published cell lines were previously negative for Mycoplasma contamination (Yamaguchi et al. Nature 2017, Hackett et al. Stem Cell Reports 2017, Hirabayashi et al. J Reprod Dev 2014). We have not further checked.
Commonly misidentified lines (See ICLAC register)	n/a

Animals and other organisms

Policy information about studies involving animals; ARRIVE guidelines recommended for reporting animal research

Laboratory animals	Described in "Animals" in the Methods section.
Wild animals	n/a
Field-collected samples	n/a
Ethics oversight	All experiments were performed in accordance with the animal care and use committee guidelines of the National Institute for Physiological Sciences.

Note that full information on the approval of the study protocol must also be provided in the manuscript.

Flow Cytometry

Plots

Confirm that:

- [x] The axis labels state the marker and fluorochrome used (e.g. CD4-FITC).
- [ ] The axis scales are clearly visible. Include numbers along axes only for bottom left plot of group (a 'group' is an analysis of identical markers).
- [ ] All plots are contour plots with outliers or pseudocolor plots.
- [x] A numerical value for number of cells or percentage (with statistics) is provided.

Methodology

Sample preparation	Hemolysed peripheral blood cells or trypsinized embryonic fibroblasts were suspended in PBS with 3% fetal bovine serum with or without DAPI.
Instrument	SH800 cell sorter (SONY)
Software	FlowJo v10
Cell population abundance	5,000 - 1,000,000 cells after excluding debris and doublets were analyzed in this study.
Gating strategy	Debris were excluded by FSC-A and SSC-A gating. Doublets were excluded by FSC-W and SSC-H gating. Dead cells were excluded by DAPI negative gating. Fluorescence negative fraction was determined using Crlj:WI rat, slc:ICR mouse or non-chimera samples.

- [ ] Tick this box to confirm that a figure exemplifying the gating strategy is provided in the Supplementary Information.
