# Supplementary figures and images for: Blastocyst complementation using Prdm14-deficient rats enables efficient germline transmission and generation of functional mouse spermatids in rats
Source: Nat Commun. 2021 Feb 26;12:1328. doi: 10.1038/s41467-021-21557-x (PMC7910474; doi:10.1038/s41467-021-21557-x)

Figure 1k

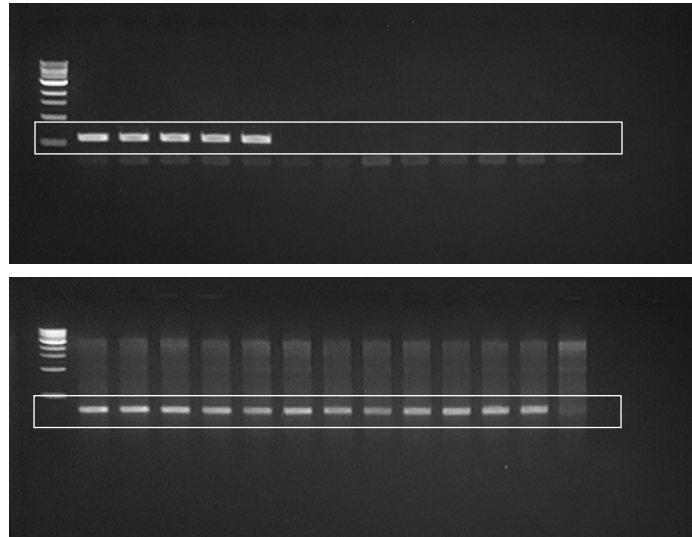

Supplementary Figure 1a

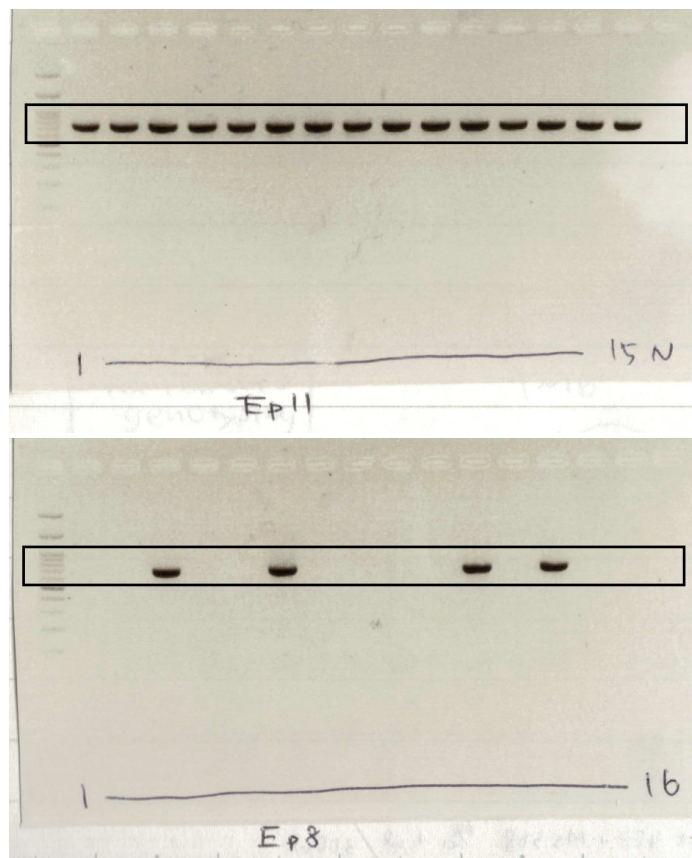

Supplementary Figure 3h

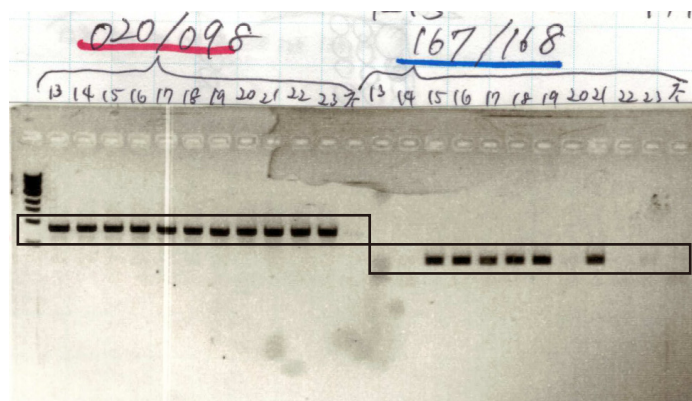

Supplement: Supplementary file 4 — Source Data [file 41467_2021_21557_MOESM4_ESM.pdf]
